# Transient activation of human cytomegalovirus lytic gene expression during latency allows cytotoxic T cell killing of latently infected cells

**DOI:** 10.1038/srep24674

**Published:** 2016-04-19

**Authors:** B. A. Krishna, B. Lau, S. E. Jackson, M. R. Wills, J. H. Sinclair, E. Poole

**Affiliations:** 1 Department of Medicine, University of Cambridge, Level 5 Laboratories Block, Addenbrooke’s Hospital, Hills Road, Cambridge CB2 0QQ.

## Abstract

Human cytomegalovirus (HCMV) latency in the myeloid lineage is maintained by repressive histone modifications around the major immediate early promoter (MIEP), which results in inhibition of the lytic viral life cycle. We now show that pharmacological inhibition of histone deacetylases (HDACs) relieves this repression of the MIEP and induces transient expression of the viral lytic immediate early (IE) antigens but, importantly, not full virus reactivation. In turn, these latently infected cells now become targets for IE-specific cytotoxic T cells (CTLs) which are present at high frequency in all normal healthy HCMV positive carriers but would normally be unable to target latent (lytic antigen-negative) cells. This approach of transiently inducing viral lytic gene expression by HDAC inhibition, in otherwise latently infected cells, offers a window of opportunity to target and purge the latent myeloid cell reservoir by making these normally immunologically undetectable cells visible to pre-existing host immune responses to viral lytic antigens.

Human cytomegalovirus (HCMV) causes significant morbidity and mortality in the immunocompromised and the immunonaïve^1^,^2^ and, as yet, an effective vaccine for the prevention of HCMV infection remains elusive^3^.

Like all herpesviruses HCMV persists for the life-time of the host after primary infection which is, at least in part, due to the consummate ability of HCMV to avoid immunosurveillance. However, this persistence is underpinned by the ability of the virus to establish a latent infection in certain cell types *in vivo*^4^ where viral genome is maintained in the absence of lytic replication. To date, all current anti-HCMV therapies target lytic virus replication; none target the latent reservoir even though reactivation from latency is a major cause of disease in many settings. Furthermore, there is increasing evidence associating HCMV persistence with long-term disease (e.g. atherosclerosis) and reactivation from latency will likely be a major contributor to this viral persistence^5^. Consequently, targeting the latent reservoir could have far reaching clinical implications.

One established site of HCMV latency *in vivo* are cells of the myeloid lineage, including CD14+ monocytes and their CD34+ progenitors^6^. In these cells, the viral MIEP, which drives lytic gene expression, is associated with repressive chromatin, which suppresses lytic transcription and maintains latent infection. Following differentiation into macrophages or dendritic cells (DCs), however, changes in the nuclear environment result in chromatin-mediated activation of the viral MIEP and reactivation of lytic replication^7^,^8^,^9^. Previous work has implicated a role for histone deacetylases (HDACs) in this differentiation-dependent regulation of MIEP activity in myeloid cells. Expression of Class I HDACs are known to decrease upon differentiation of monocytes to macrophages^10^, consistent with the known changes in repressive and activatory histone markers on the MIEP during latency and reactivation in myeloid cells^6^,^7^,^10^,^11^,^12^. Similarly, other changes in cellular gene expression are known to occur during HCMV latency to aid latent carriage^13^,^14^,^15^ and this also includes changes in cellular miRNAs. One of these, miRNA hsa-miR206 which decreases during latent infection of CD34+ cells^15^, is known to target a class II HDAC, HDAC4^16^ and consistent with this, we now show that latent infection is associated with an increase in this cellular HDAC.

In the context of an inadequate immune response (either in transplant patients, the immunocompromised or the immunonaïve), these reactivation events can result in virus dissemination to multiple target organs and subsequent clinical disease^1^.

Importantly, we show here that the treatment of latently infected cells with the Class II -specific HDAC inhibitor (HDACi) MC1568 leads to the transient expression of viral lytic gene products which then makes these cells novel targets for pre-existing HCMV-specific CTLs, routinely present at high frequency in all immunocompetent virus carriers. These observations are proof of principle that the use of HDAC inhibitors can result in removal of latently infected cells which may be a useful novel therapeutic strategy in certain clinical settings.

## Results and Discussion

### Treatment of latently infected monocytes with the HDAC4 inhibitors leads to a transient induction of IE gene expression

**Figure 1:**
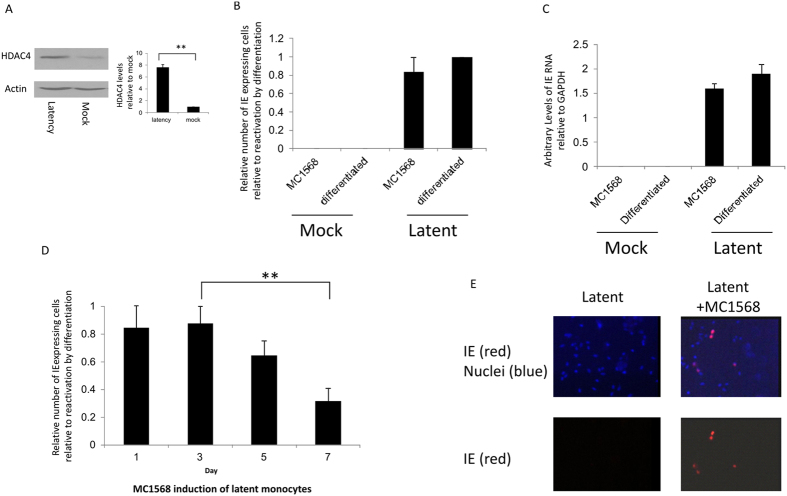
HDAC4 is increased during latency and targeted by MC1568 to induce lytic gene expression. 5 day latent (latency) or control (mock) monocytes were analysed for actin or HDAC4 by Western blot. Samples were run on the same gel and autoradiographs from different exposures were cropped to show the HDAC4 and actin. Blots were analysed using Image J freeware in triplicate. Standard deviations are shown and statistical significance was determined using the Student’s t-test (**A**). 5 day latent monocytes (latent) or control (mock) monocytes were treated for 24 h with MC1568 or differentiation media. Following 24 h of treatment with MC1568 or 7 day differentiation, cells were stained for IE expression. Data shown (6 replicates) are relative to the level of IE induced following reactivation by differentiation (**B**) or harvesting for RT-qPCR analysis (**C**). 5 day latent monocytes were treated with MC1568 and analysed for IE expression after 24 h (Day 1) and then at Day 3, 5 and 7 without removal of MC1568 from the media. Data shown (6 replicates) are relative to the level of IE induced following reactivation by differentiation. Standard deviations are shown and statistical significance was determined using the Student’s t-test (**D**). 5 day latent monocytes were either untreated (latent) or treated with MC1568 for 24 h (latent plus MC1568) then fixed and stained for IE expression (red) with nuclei shown in blue (**E**).

We have previously shown that latent infection of myeloid cells results in major changes in cellular gene expression^14^ as well as changes in cellular microRNAs^15^. One cellular microRNA (miRNA) which was identified as being decreased during latency is hsa-miR-206^15^, which has previously been shown to target HDAC4^16^. Consistent with this, latent infection of monocytes with HCMV did indeed result in elevated levels of HDAC4 (Fig. 1A). Furthermore, the MIEP was found to be heavily associated with HDAC4 during latent infection (Supplementary Figure 1) suggesting a suppressive role for HDAC4 in maintaining latency.

We reasoned, therefore, that induction of histone deacetylases by latent virus may be important for the latency-associated repression of viral lytic gene expression and that deacetylase inhibition might impact on this regulation of viral gene expression during latency. Consequently, we tested whether inhibition of HDAC4 during latent infection had any effect on IE gene expression using the Class II-specific HDAC inhibitor MC1568, which is known to inhibit HDAC4 activity *in vivo* and *in vitro*^17^. Figure 1B–D show that treatment of latently infected monocytes with MC1568 (at non-toxic concentration, supplementary Figure 2A,B) resulted in a robust induction of IE lytic gene expression at levels of both IE protein (graphically represented in Fig. 1B and shown by immunofluorescence in Fig. 1E) and RNA (Fig. 1C) which waned when drug was removed (Fig. 1D).

**Figure 2:**
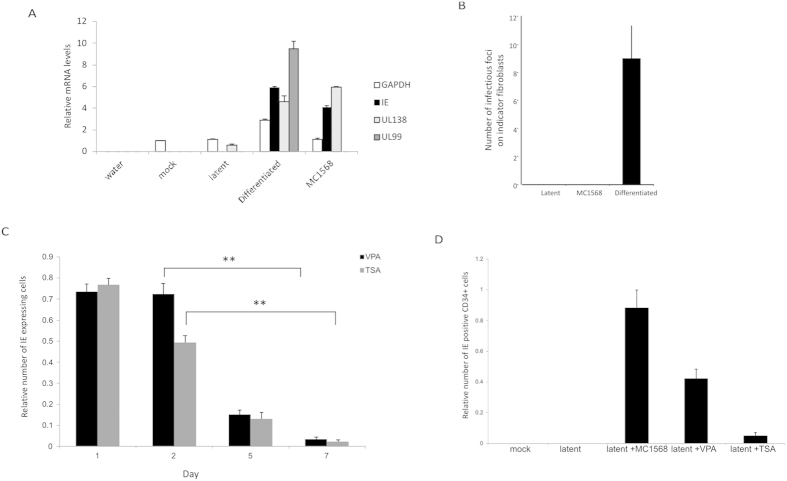
MC1568 does not cause full reactivation and pan-HDAC inhibitors also induce IE expression. 5 day latent (latent) monocytes were left untreated, treated with MC1568 for 7 days or differentiated. RNAs encoding viral IE, UL99 (pp28), UL138 and cellular GAPDH were analysed in duplicate by RT-qPCR and standard deviations are shown (**A**). Alternatively, 5 day latent monocytes (latent) were treated with MC1568 or differentiated and then co-cultured with fibroblasts to analyse infectious foci formation (**B**). 5 day latent monocytes were also treated over 1–7 days with VPA or TSA and analysed for IE expression. Data shown (6 replicates) are relative to the level of IE induced following reactivation by differentiation (**C**). Additionally, 5 day latent CD34+ cells (latent) were treated with MC1568, TSA or VPA for 24h (Day 1) and levels of IE expression were also analysed at days 2, 5 and 7 without removal of the drugs. Data are shown (6 replicates) relative to IE expression induced by differentiation of CD34+ cells. Mock represents IE expression in uninfected cells. Standard deviations are shown and statistical significance was determined using the Student’s t-test (**D**).

Interestingly, this induction of viral IE gene expression by MC1568 did not result in reactivation of the full lytic cycle, even after 7 days continuous treatment, as no expression of viral UL99 (pp28), a true late viral gene only expressed after viral DNA replication, was detected (Fig. 2A). Consistent with this, MC1568-treated monocytes showed no evidence of reactivation of infectious virus as detected by co-culture on indicator fibroblasts (Fig. 2B). Additionally, if latently infected monocytes were treated with other pan-specific HDAC inhibitors such as valproic acid (VPA) or trichostatin A (TSA), a transient induction of IE gene expression was also detectable (Fig. 2C). Interestingly, treatment of latently infected CD34+ progenitor cells with VPA and MC1568 also induced IE gene expression (Fig. 2D). However, consistent with reports that CD34+ cells are unresponsive to TSA^18^, induction of IE gene expression from latently infected CD34+ cells was not observed if cells were induced with TSA (Fig. 2D).

Therefore, importantly, these cells - expressing IE antigens but stalled in full reactivation -would be prime targets for the well established immunodominant IE-specific T cell responses known to be at high frequency in normal HCMV carriers.

### Transient expression of lytic IE antigen in HDAC-inhibitor treated cells opens a window of opportunity to target latent cells with IE-specific CTLs

**Figure 3:**
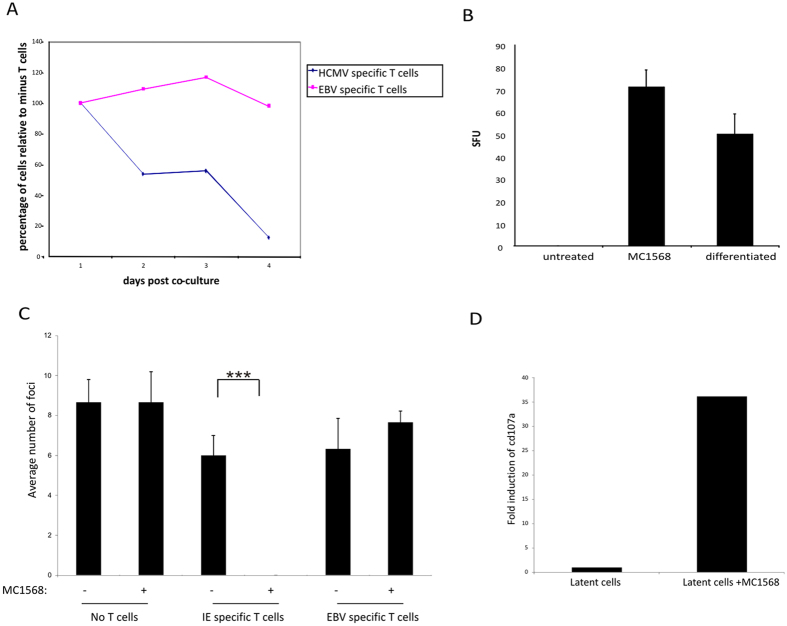
Treatment of experimentally latent monocytes with MC1568 or VPA reduces reactivation mediated by IE-specific T cells. Monocytes latently infected with an IE-GFP tagged virus for 5 days were treated with MC1568 and cultured with IE or EBV-specific T cells, following which, monocytes expressing IE-GFP were counted. The graph shows numbers of IE-GFP expressing monocytes relative to IE expression in the absence of T cells (**A**). Alternatively, 5 day latent monocytes (untreated) were treated with MC1568 or induced to differentiate (differentiated), cultured with IE-specific T cells and analysed by IFN-gamma ELISpot. The graph represents the number of spot forming units (SFUs) above background (**B**). Additionally, 5 day latent monocytes with (+) or without (−) MC1568 treatment were co-cultured without (no T cells) or with IE-specific T cells or EBV-specific T cells. After this, monocytes were reactivated by differentiation and infectious foci quantified by co-culture on indicator fibroblasts (**C**). 5 day latent monocytes (latent) monocytes were left untreated (latent) or treated with MC1568 (+MC1568) and co-cultured with IE or EBV-specific T cells, then analysed for CD107a expression. The graph represents fold induction of CD107a expression in IE1-specific T cells after presentation to latently infected cells treated with MC1568 compared to untreated latently infected cells. Values were corrected to CD107a expression following presentation of the same MC1568 treated and untreated latent cells to EBV specific (irrelevant) T cells (**D**).

Between 0.5–10% of total circulating CD8+ CTLs in healthy HCMV seropositive donors are specific for the viral major IE lytic gene^19^. However, these IE-specific T cells do not target latently infected (lytic antigen negative) cells; consistent with the fact that latent infection is not cleared despite such high populations of functional IE-specific CTLs. We predicted, however, that treatment of latently infected cells with MC1568 should make them become IE-specific CTL targets. Figure 3A shows that co-culture of MC1568-treated latent monocytes with IE-specific T cell clones, but not irrelevant EBV-specific T cells, resulted in a robust decrease in the number of monocytes positive for IE gene expression. Consistent with the mediation of this effect through T cell effector function, MC1568-treated monocytes also resulted in increased IFN-gamma responses by HCMV-specific T cells to levels comparable to monocytes induced to reactivate by full differentiation (Fig. 3B). Perhaps more importantly, MC1568-treated monocytes, after co-culture with IE-specific CTLs, showed a robust reduction in their ability to reactivate virus after standard differentiation as detected by re-infection of co-cultured indicator fibroblasts (Fig. 3C).

Although the data, so far, suggest that the IE-specific CTLs were recognising and killing the latently infected cells, which had been induced to express IE antigens by HDAC inhibitor treatment, it is possible that this removal of monocytes expressing IE antigen resulted from CTL-mediated repression of IE gene expression and that these monocytes still carried reactivatable virus. In order to confirm that the disappearance of monocytes transiently expressing IE was due to T cell killing, rather than perhaps re-repression of IE gene expression, we confirmed the expression of CD107a (LAMP-1 an indication of cytotoxic T cell degranulation and part of the CTL killing mechanism) in these IE-specific T cells after surveillance of MC1568-treated latent monocytes (Fig. 3D). Figure 3D shows that CD107a was indeed upregulated in T cells which had been co-cultured with latent monocytes that had been induced to express IE by MC1568 and, therefore, these IE-specific CTLs were not only recognising but also released their cytotoxic granules, an indication of killing activity. Taken together, these data clearly showed that HDAC inhibition results in transient induction of viral IE gene expression in experimentally latent monocytes, resulting in them becoming temporarily visible to IE-specific CTLs.

**Figure 4:**
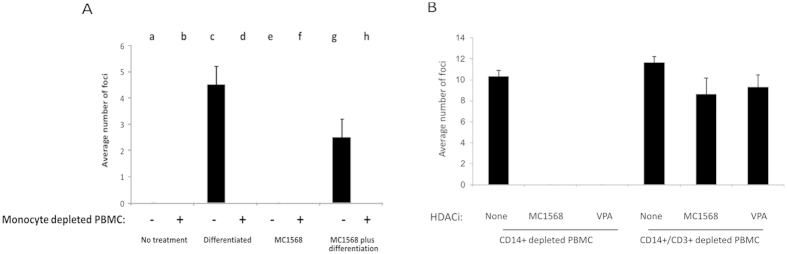
Treatment of naturally latent monocytes with MC1568 or VPA reduces reactivation. Monocytes from PBMCs of HCMV seropositive individuals were isolated by plastic adherence. Adherent monocytes were left untreated (no treatment, columns a and b), differentiated to reactivate virus (differentiated, columns c and d), treated with MC1568, (columns e and f) and cultured in the presence (+) or absence (−) of the residual PBMCs (after monocyte depletion). Additionally, wells of MC1568-treated monocytes, after removal of T cells, were also subsequently induced to differentiate (columns g and h). In all cases, any reactivated virus was quantified by fibroblast co-culture and staining for IE positive foci (**A**). Finally, CD14+ monocytes from PBMCs of two HCMV seropositive individuals were isolated, adhered to plastic and left untreated (none), treated with MC1568 or VPA for 24 h. The PBMCs left after CD14+ isolation were either left untreated (CD14+ depleted PBMC) or further depleted of T cells by CD3+ positive selection (CD14+/CD3+ depleted PBMCs). Subsequently, PBMCs were removed by washing and cells were differentiated and any reactivated virus was quantified by fibroblast co-culture and staining for IE foci (**B**). Standard deviations are shown and statistical significance was determined using the Student’s t-test.

However, a key question is whether drug treatment permits naturally latently infected cells in the peripheral blood of healthy donors to become targetable by blood resident HCMV-specific CTLs. To address this we carried out analyses on total peripheral blood mononuclear cells (PBMC) from two seropositive individuals to allow assessment of the effectiveness of MC1568 in clearing naturally latent HCMV from the peripheral blood. Figure 4A shows that naturally latent cells do not produce infectious virus (column a), as expected (shown by a lack of infectious foci on indicator fibroblasts). Again, as expected, latent cells induced to differentiate, reactivate infectious virus (column c) but this reactivation is ablated in the presence of T cells (column d). This argues that the T cells in the PBMC compartment are able to detect differentiation-dependent virus reactivation. Consistent with the fact that MC1568 treatment does not induce full virus reactivation in latently infected monocytes, HDACi treatment showed no virus production regardless of whether they were cultured with or without autologous T cell-containing PBMCs (columns e and f). However, autologous T cell-containing PBMCs completely ablate the ability of these transiently IE1-expressing latent cells to reactivate endogenous latent virus after their subsequent differentiation (columns g and h). Repeating the analysis using T cell-depleted PBMCs confirmed that the T cell population in these PBMCs were effecting this reduction in latent cells capable of reactivating infectious virus (Fig. 4B).

Taken together, these data argue that pre-existing autologous IE-specific T cells in the peripheral blood compartment of healthy HCMV carriers are able to target and purge naturally latently infected monocytes after these monocytes have been treated to induce IE expression transiently with HDAC inhibitors.

Our view is that such novel immunotherapeutic strategies could lend themselves to, for example, the treatment of stem cell donations from HCMV seropositive donors to reduce latent load prior to engraftment but, as a number of HDAC inhibitors are already in clinical use, could also be used in donors prior to donation.

## Materials and Methods

### Viruses, cells and latency

Cell isolations and establishment of experimental latency using HCMV TB40E or IE2-EYFP-tagged TB40E have been described previously^14^. For the studies of natural latency, approximately 5 × 10^8^ CD14+ monocytes were isolated as described^14^ but using apheresis cones from seropositive individuals. Frozen CD34+ progenitor cells were obtained from Lonza.

### Quantification of experimental and natural latency

All assays quantifying IE reactivation in response to treatments were normalised to the number of IE positive cells following reactivation by differentiation of CD14+ or progenitor cells into dendritic cells as described previously^20^. In our experiments, experimental latency was routinely established in approximately 20% of cells. Reactivation of IE expression by differentiation varied from 0.25–25% of these latent cells. Therefore, in a well of 10^5^ cells, the number of IE positive cells following reactivation routinely ranged from 5 to 500 IE expressing cells per well between experiments. Consequently, data were normalised to numbers of IE-expressing cells after differentiation.

For natural latency, differentiation of approximately 10^6^ cells routinely resulted in 2–10 IE positive foci of infection and these numbers are directly graphically represented. Unless otherwise stated, each experiment was carried out a minimum of two times with at least duplicate samples.

### Statistics

Error bars represent standard deviation and P values were determined using the Student’s t-test. Values are represented by the standard asterisk system where P < 0.05* is statistically significant; P < 0.01** is of medium significance and P < 0.001*** is statistically highly significant.

### Western blotting

Western blotting was carried out as described previously^21^ with the primary antibodies rabbit anti-actin (Abcam) and mouse anti-HDAC4 (Cell Signalling).

### Immunofluoresence

Cells were fixed and stained as described previously^22^. Cells were stained with mouse anti-IE (Argene 11-003) followed by goat anti-mouse (Alexafluor 633 or 488, Molecular Probes) with Hoechst nuclear stain. Alternatively, if cells were infected with YFP-IE TB40E^23^, nuclei were stained in live cells with Hoechst.

### T cell co-culture

CD14+ monocytes were plated at 1 × 10^5^ per well of a 96 well plate and then co-cultured in *X-Vivo* 15 supplemented with 2 mM L-Glutamine with donor-matched (HLA-A2) EBV or HCMV-specific CD8+ T cells at a ratio of 5:1. The number of HCMV IE-expressing monocytes were enumerated over several days as indicated.

### ELISpot

ELISPOT plates were prepared, coated and blocked according to manufacturer’s instruction (eBioscience). CD14+ monocytes, isolated directly *ex vivo* were isolated from total PBMC by magnetic activated cell sorting (MACS), were co-cultured with donor-matched IE-specific CD8+ T cells in 100 μl RPMI-10 per well (of a 96 well Multiscreen IP sterile plate (Millipore, UK)). Plates were incubated for 48 hours at 37 °C in 5% CO_2_, and developed according to manufacturer’s instructions. Plates were read using an ELISPOT plate scanner (ELISPOT Reader System, AID) and spots enumerated using ImageJ (National Institutes of Health).

### CD107a assay

Cells were co-incubated with IE-specific CD8+ T cell lines or EBV-specific T cell lines overnight in the presence of CD107a Alexa fluor 647, 5 μg/ml Brefeldin A and 2μM Monensin (all from BioLegend) at 37 °C in a humidified CO_2_ atmosphere. CD8+ T cells were harvested and washed, then stained with a combination of surface antibodies (Supplementary Table 1A) and LIVE/DEAD Fixable Yellow Dead cell stain (Invitrogen) at 4 °C. Cells were fixed and permeabilised using FIX&PERM (ADG, Kaumberg, Austria) and stained intracellularly with antibodies (Supplementary Table 1B) at 4 °C in the dark. Samples were washed and fixed in a final 1% paraformaldehyde solution and acquired on a BD LSR Fortessa cytometer using FACSdiva software (BD Biosciences). Data was analysed using FlowJo software (Treestar). Responding CD8+ T cell populations were identified by the expression of CD107a above the background expression observed in the mock infected control following elimination of doublets, removal of monocytes and B cells and dead cells from the analysed population.

### Drug treatments

MC1568 (Sigma) was tested for cytotoxicity (Supplementary Figure 1) and used at the non-toxic concentration of 1000nM. Trychostatin A (TSA) (New England Biolabs), valproic acid (VPA) (Miltenyi) were made up as directed by the manufacturer and used at the concentrations indicated

### RT-qPCR

RNAs were isolated using the miRNeasy mini-kit and amplified using the Quantitect virus + ROX virus kit (Qiagen). The viral IE transcript was amplified alongside the housekeeping transcript GAPDH using the following primers and probes: IE: CAAGAACTCAGCCTTCCCTAAGAC and TGAGGCAAGTTCT**GC**AATGC with the probe [FAM]CCAATGGCTGCAGTCAGGCCATG[TAM] and GAPDH GGAAGCTTGTCATCAATG and CCCCACTTGATTTTGGAG with the probe [JOE]ATCACCATCTTCCAGGAGCGAG[BHQ1]. Viral transcript UL138 was detected with primers CGCTGTTTCTCTGGTTAG and CAGACGATACCGTTTCTC with probe [Cy5]CCGACGACGAAGACGATGAAC[BHQ2] and viral transcript UL99 (pp28) with the primers CGAACTCTGCAAACGAATA and GAGGGATGTTGTCGTAGG with the probe [Cy3]CGTAGAGACACCTGGCGACC[BHQ2]. Samples were analysed with an ABI 7500 Fast Real Time machine using MicroAmp Fast Optical 96 well reaction plates using the RT parameters: 50 °C for 20 min followed by heat inactivation 95 °C for 5 min then PCR steps: 50 cycles, 95 ^o^C for 15 s and 60 ^o^C for 45 s. Data were accrued from reactions carried out in the presence and absence of RT and the minus RT values subtracted from the plus RT samples to obtain the RNA levels. All primers were tested against a standard curve to allow comparison between samples.

### ChIP and qPCR

Chromatin immunopreciptition assays were carried out as described previously^24^. Lysates were incubated with either an antibody to HDAC4 (1 μg/ml Santa Cruz Biotechnology) or the isotype-matched control (1μg/ml). Following elution of immunoprecipitated DNA, the MIEP was amplified by qPCR using the Quantitect virus + ROX virus kit (Qiagen) with the following primers and probe: CCAAGTCTCCACCCCATTGAC and GACATTTTGGAAAGTCCCGTTG with the probe [FAM]TGGGAGTTTGTTTTGGCACCAAA[TAM] and with the following PCR parameters: 50 cycles. 95 ^o^C for 15 s and 60 ^o^C for 45 s.

### Cytotoxicity assays

To analyse the levels of cell death, cells were stained directly with trypan blue in PBS (a live dead discriminator). Alternatively, the viability of cells was determined using the LIVE/DEAD Fixable dead cell stain kit (Invitrogen, Molecular probes) and analysed by FACS.

#### Ethics statement

All human samples were obtained under ethical approval and after approval of protocols from the Cambridgeshire 2 Research Ethics Committee (REC reference 97/092) and that these protocols were conducted in accordance with the Declaration of Helsinki. Informed written consent was obtained from all of the volunteers included in this study prior to providing blood samples and all experiments were carried out in accordance with the approved guidelines.

## Additional Information

**How to cite this article**: Krishna, B. A. *et al*. Transient activation of human cytomegalovirus lytic gene expression during latency allows cytotoxic T cell killing of latently infected cells. *Sci. Rep*. **6**, 24674; doi: 10.1038/srep24674 (2016).

## Supplementary Material

Supplementary Information

## References

[b1] Alford C. A., Stagno S. & Pass R. F. Natural history of perinatal cytomegaloviral infection. Ciba Found Symp, 77, 125–147 (1979). 233356 10.1002/9780470720608.ch9

[b2] Fishman J. A. Infections in immunocompromised hosts and organ transplant recipients: essentials. Liver Transpl 17 Suppl 3, S34–37 (2013). 21748845 10.1002/lt.22378

[b3] Khanna R. & Diamond D. J. Human cytomegalovirus vaccine: time to look for alternative options. Trends Mol Med 12, 26–33 (2006). 16337831 10.1016/j.molmed.2005.11.006

[b4] Hahn G., Jores R. & Mocarski E. S. Cytomegalovirus remains latent in a common precursor of dendritic and myeloid cells. Proc Natl Acad Sci USA 95, 3937–3942 (1998). 9520471 10.1073/pnas.95.7.3937 PMC19941

[b5] Popovic M. . Human cytomegalovirus infection and atherothrombosis. J Thromb Thrombolysis 33, 160–172 (2013). 22161772 10.1007/s11239-011-0662-x

[b6] Sinclair J. & Poole E. Human cytomegalovirus latency and reactivation in and beyond the myeloid lineage. Future Virology 6, 7 (2014). 10.1016/j.jcv.2007.11.014 18164651

[b7] Reeves M. B., MacAry P. A., Lehner P. J., Sissons J. G. & Sinclair J. H. Latency, chromatin remodeling, and reactivation of human cytomegalovirus in the dendritic cells of healthy carriers. Proc Natl Acad Sci USA 102, 4140–4145 (2005). 15738399 10.1073/pnas.0408994102 PMC554799

[b8] Reeves M. . Autorepression of the human cytomegalovirus major immediate-early promoter/enhancer at late times of infection is mediated by the recruitment of chromatin remodeling enzymes by IE86. J Virol 80, 9998–10009 (2006). 17005678 10.1128/JVI.01297-06 PMC1617317

[b9] Poole E. . Alveolar macrophages isolated directly from HCMV seropositive individuals are sites of HCMV reactivation *in vivo*. J Infect Dis, doi: 10.1093/infdis/jiu837 (2014). PMC4442624 25552371

[b10] Murphy J. C., F. W., Verdin E. & Sinclair J. H. Control of cytomegalovirus lytic gene expression by histone acetylation. EMBO J. 21, 1112–1120 (2002). 11867539 10.1093/emboj/21.5.1112 PMC125876

[b11] Reeves M. B., Lehner P. J., Sissons J. G. & Sinclair J. H. An *in vitro* model for the regulation of human cytomegalovirus latency and reactivation in dendritic cells by chromatin remodelling. J Gen Virol 86, 2949–2954 (2005). 16227215 10.1099/vir.0.81161-0

[b12] Poole E., Wills M. & Sinclair J. Human Cytomegalovirus Latency: Targeting Differences in the Latently Infected Cell with a view to Clearing Latent Infection. New Journal of Science 2014, 10 (2014).

[b13] Mason G. M., Poole E., Sissons J. G., Wills M. R. & Sinclair J. H. Human cytomegalovirus latency alters the cellular secretome, inducing cluster of differentiation (CD)4+ T-cell migration and suppression of effector function. Proc Natl Acad Sci USA 109, 14538–14543 (2012). 22826250 10.1073/pnas.1204836109 PMC3437838

[b14] Weekes M. P. . Latency-associated degradation of the MRP1 drug transporter during latent human cytomegalovirus infection. Science 340, 199–202 (2013). 23580527 10.1126/science.1235047 PMC3683642

[b15] Poole E., McGregor Dallas S. R., Colston J., Joseph R. S. & Sinclair J. Virally induced changes in cellular microRNAs maintain latency of human cytomegalovirus in CD34 progenitors. J Gen Virol 92, 1539–1549 (2011). 21471310 10.1099/vir.0.031377-0

[b16] Winbanks C. E. . TGF-beta regulates miR-206 and miR-29 to control myogenic differentiation through regulation of HDAC4. J Biol Chem 286, 13805–13814 (2011). 21324893 10.1074/jbc.M110.192625 PMC3077581

[b17] Nebbioso A. . Selective class II HDAC inhibitors impair myogenesis by modulating the stability and activity of HDAC-MEF2 complexes. EMBO Rep 10, 776–782 (2009). 19498465 10.1038/embor.2009.88 PMC2693879

[b18] Travers H., Spotswood H. T., Moss P. A. & Turner B. M. Human CD34+ hematopoietic progenitor cells hyperacetylate core histones in response to sodium butyrate, but not trichostatin A. Experimental cell research 280, 149–158 (2002). 12413881 10.1006/excr.2002.5632

[b19] Jackson S. E., Mason G. M. & Wills M. R. Human cytomegalovirus immunity and immune evasion. Virus Res 157, 151–160 (2013). 21056604 10.1016/j.virusres.2010.10.031

[b20] Poole E., Reeves M. & Sinclair J. H. The use of primary human cells (fibroblasts, monocytes, and others) to assess human cytomegalovirus function. Methods Mol Biol 1119, 81–98, doi: 10.1007/978-1-62703-788-4_6 (2014). 24639219

[b21] Poole E. . The myeloid transcription factor GATA-2 regulates the viral UL144 gene during human cytomegalovirus latency in an isolate-specific manner. J Virol 87, 4261–4271 (2013). 23365437 10.1128/JVI.03497-12 PMC3624344

[b22] Poole E., King C. A., Sinclair J. H. & Alcami A. The UL144 gene product of human cytomegalovirus activates NFkappaB via a TRAF6-dependent mechanism. Embo J 25, 4390–4399 (2006). 16932746 10.1038/sj.emboj.7601287 PMC1570428

[b23] Straschewski S. . Human cytomegaloviruses expressing yellow fluorescent fusion proteins--characterization and use in antiviral screening. PLoS One 5, e9174 (2010). 20161802 10.1371/journal.pone.0009174 PMC2820100

[b24] Poole E. . NF-kappaB-mediated activation of the chemokine CCL22 by the product of the human cytomegalovirus gene UL144 escapes regulation by viral IE86. J Virol 82, 4250–4256 (2008). 18287226 10.1128/JVI.02156-07 PMC2293074

