# Supplementary material for: Transient activation of human cytomegalovirus lytic gene expression during latency allows cytotoxic T cell killing of latently infected cells
Source: Sci Rep. 2016 Apr 19;6:24674. doi: 10.1038/srep24674 (PMC4835774; doi:10.1038/srep24674)
Supplement: Supplementary Information [file srep24674-s1.docx]

Transient activation of human cytomegalovirus lytic gene expression

during latency allows cytotoxic T cell killing of latently infected cells

**Authors:** B. Krishna^2^, B. Lau^2^, S.E. Jackson^2^, M.R. Wills^2^, J.H. Sinclair^12^* and E. Poole^12^*

**Affiliations:**

^2^Department of Medicine, University of Cambridge, Level 5 Laboratories Block, Addenbrooke’s Hospital, Hills Road, Cambridge CB2 0QQ.

Supplementary figure 1

HDAC4 is associated with the MIEP during latency. Monocytes in which latency had been established for 5 days were analysed directly for MIEP DNA by qPCR (input) or were analysed by ChIP for association with HDAC4. Standard deviation error bars are presented

Supplementary Figure 2





MC1568 is not toxic to myeloid cells. Monocytes were treated with increasing concentrations of MC1568 for 24h before trypan blue analysis (A) or FACS analysis using molecular probes live/dead discriminator (B).

Supplementary Table 1A

| **Antibody** | **Manufacturer** |
| --- | --- |
| CD3 brilliant violet 650 | BioLegend |
| CD14 FITC | eBiosciences |
| CD19 FITC | eBiosciences |

Supplementary Table 1B

| **Antibody** | **Manufacturer** |
| --- | --- |
| CD8 AlexaFluor 700 | BioLegend |
| IFNgammaPE | BD Biosciences |
